# SMAD4 Positive Pancreatic Ductal Adenocarcinomas Are Associated with Better Outcomes in Patients Receiving FOLFIRINOX-Based Neoadjuvant Therapy

**DOI:** 10.3390/cancers15153765

**Published:** 2023-07-25

**Authors:** Marie-Lucie Racu, Dana Bernardi, Aniss Chaouche, Egor Zindy, Julie Navez, Patrizia Loi, Calliope Maris, Jean Closset, Jean-Luc Van Laethem, Christine Decaestecker, Isabelle Salmon, Nicky D’Haene

**Affiliations:** 1 Departement of Pathology, CUB Hôpital Erasme, Hôpital Universitaire de Bruxelles (H.U.B), Université Libre de Bruxelles (ULB), Route de Lennik 808, 1070 Brussels, Belgium; dana.bernardi@hubruxelles.be (D.B.); aniss.chaouche@ulb.be (A.C.); calliope.maris@hubruxelles.be (C.M.); isabelle.salmon@hubruxelles.be (I.S.); nicky.dhaene@hubruxelles.be (N.D.); 2 Digital Image Analysis in Pathology (DIAPath), Center for Microscopy and Molecular Imaging (CMMI), Université Libre de Bruxelles (ULB), 6041 Gosselies, Belgium; egor.zindy@ulb.be (E.Z.); christine.decaestecker@ulb.be (C.D.); 3 Laboratory of Image Synthesis and Analysis (LISA), Brussels School of Engineering/École Polytechnique de Bruxelles, Université Libre de Bruxelles (ULB), 1050 Brussels, Belgium; 4 Department of Hepato-Biliary-Pancreatic Surgery, CUB Hôpital Erasme, Hôpital Universitaire de Bruxelles (H.U.B), Université Libre de Bruxelles (ULB), Route de Lennik 808, 1070 Brussels, Belgium; julie.navez@hubruxelles.be (J.N.); patrizia.loi@hubruxelles.be (P.L.); jean.closset@hubruxelles.be (J.C.); jean-luc.vanlaethem@hubruxelles.be (J.-L.V.L.)

**Keywords:** pancreas, pancreatic ductal adenocarcinoma, SMAD4, neoadjuvant therapy, prognosis, biomarker, overall survival, progression-free survival, metastasis-free survival

## Abstract

**Simple Summary:**

Patients with pancreatic ductal adenocarcinoma (PDAC) are increasingly receiving neoadjuvant therapy (NAT) with the aim of improving patient care. However, complete tumor regression remains rare, and there is a dire need for biomarkers that can predict responses to NAT. SMAD4 is a protein that is inactivated in about half of PDACs and has been described as associated with metastasis and resistance to therapy. The aim of our study is to investigate whether SMAD4 status is a prognostic and predictive factor in patients with pancreatic cancer receiving NAT. In our cohort of patients, SMAD4 loss was associated with quicker progression and metastasis development. When patients were subgrouped depending on their type of neoadjuvant treatment, those receiving FOLFIRINOX-based NAT and presenting SMAD4-positive tumors showed the best outcome. This result suggests that SMAD4 may be a potential biomarker for patients with pancreatic cancer receiving NAT and may help improve patient care.

**Abstract:**

Background: SMAD4 is inactivated in 50–55% of pancreatic ductal adenocarcinomas (PDACs). SMAD4 loss of expression has been described as a negative prognostic factor in PDAC associated with an increased rate of metastasis and resistance to therapy. However, the impact of SMAD4 inactivation in patients receiving neoadjuvant therapy (NAT) is not well characterized. The aim of our study was to investigate whether SMAD4 status is a prognostic and predictive factor in patients receiving NAT. Methods: We retrospectively analyzed 59 patients from a single center who underwent surgical resection for primary PDAC after NAT. SMAD4 nuclear expression was assessed by immunohistochemistry, and its relationship to clinicopathologic variables and survival parameters was evaluated. Interaction testing was performed between SMAD4 status and the type of NAT. Results: 49.15% of patients presented loss of SMAD4. SMAD4 loss was associated with a higher positive lymph node ratio (*p* = 0.03), shorter progression-free survival (PFS) (*p* = 0.02), and metastasis-free survival (MFS) (*p* = 0.02), but it was not an independent prognostic biomarker in multivariate analysis. Interaction tests demonstrated that patients with SMAD4-positive tumors receiving FOLFIRINOX-based NAT showed the best outcome. Conclusion: This study highlights the potential prognostic and predictive role of SMAD4 status in PDAC patients receiving FOLFIRINOX-based NAT.

## 1. Introduction

Pancreatic ductal adenocarcinoma (PDAC) represents 85–90% of solid pancreatic tumors and is one of the most lethal solid cancers, with a five-year survival of only 11%, all stages confounded [1,2]. Currently, it is the seventh cancer showing the highest estimated age-standardized mortality rate in the world and is predicted to become the second leading cause of cancer-related death before 2030 in the USA [2,3,4]. PDAC may, therefore, become a real problem of public health due to its rise in incidence, particularly in Europe and North America.

Thirty to thirty-five percent of PDAC patients will present with borderline resectable or locally advanced pancreatic cancer (BRPC and LAPC, respectively) at diagnosis [5,6]. At the present time, the only curative treatment for PDAC is surgery, but only 10–20% of patients are eligible at the moment of diagnosis [2,5,6,7].

The current standard of care for resectable PDAC is upfront surgery with six months of adjuvant therapy administered in the 12 weeks after tumor resection [2]. However, even after curative treatment, incomplete resection (R1/R2) can be observed in 17–85% of cases [8]. Moreover, the recurrence rate for PDAC is extremely high. An autopsy series of patients with PDAC showed that 77–88% indicated distant metastasis or local progression [9,10]. Furthermore, 60% of patients with upfront PDAC curative surgery are not able to receive adjuvant treatment due to postoperative complications, increasing the risk of recurrence after surgery [11].

In an attempt to achieve complete resection, patients with PDAC are increasingly being treated with neoadjuvant therapy (NAT). Indeed, it has been postulated that NAT may increase tumor downstaging and R0 resection rates, decrease lymph node (LN) metastasis, reduce post-operative complications, and raise median survival [2,7,12].

However, PDAC remains a largely treatment-refractory cancer: only 1–10% of patients receiving NAT show complete pathological response [12,13,14,15]. Thus, there is a dire need for predictive and prognostic biomarkers for PDAC patients receiving NAT.

PDAC’s genomic landscape has been extensively studied over the years [16,17]. It is characterized by four main gene alterations: mutations in the oncogene KRAS and inactivation of the tumor suppressor genes CDKN2A, TP53, and SMAD4 [17].

SMAD4 is inactivated in 50–55% of PDACs [18,19]. SMAD4 is a member of the SMAD protein family and is the central mediator of the TGF-beta signaling pathway. When activated, the TGF-beta receptor phosphorylates the proteins SMAD2/3 which can then form a protein heterocomplex with SMAD4 in the cytoplasm. This complex can then translocate into the nucleus and regulate the expression of genes implicated in cell survival, proliferation, microenvironment modulation, immunoregulation, and cell migration. SMAD4 is inactivated mainly by two mechanisms in PDAC: homozygous deletion or a mutation associated with the loss of the other allele [20,21]. SMAD4 gene inactivation can be reliably assessed by immunohistochemistry, regardless of the type of alteration [22].

Loss of SMAD4 expression has been shown to be associated with shorter overall survival (OS), disease-free survival (DFS) [23,24,25], increased metastasis [9,24,26], and resistance to therapy [27,28,29]. However, SMAD4′s role has not been well characterized in PDAC patients receiving NAT. The few existing studies suggest that SMAD4 loss is associated with shorter survival, worse tumor regression scores (TRS), and metastatic progression under NAT [30,31,32].

The aim of this study was to investigate whether SMAD4 status could be a potential prognostic and predictive biomarker for pancreatic cancer patients receiving NAT that could possibly optimize patient care.

## 2. Materials and Methods

For this study, the reporting recommendations for tumor marker prognostic studies (REMARK) were followed [33].

### 2.1. Clinical Series

We retrospectively analyzed the data of 59 patients who underwent surgical resection for a primary PDAC at Erasme University Hospital (Brussels, Belgium) from January 2004 to September 2021. We included adult patients with no recorded metastasis at the moment of surgery and with a follow-up of at least one month. Acinar cell carcinoma, intraductal papillary mucinous neoplasm, mucinous cystic neoplasm, neuroendocrine tumors, or mixed neuroendocrine-non-neuroendocrine neoplasms were excluded from the analysis.

Data including clinical (sex, age, pre-operative CA19.9 blood levels, resectability at diagnosis, year of surgery, and type of neoadjuvant and adjuvant therapy) and histopathological (histological grading, size, pT, pN, positive lymph node (LN) ratio, lymphovascular, and perineural invasion and TRS) were collected for all cases and can be found in Table 1. Formalin-fixed and paraffin-embedded (FFPE) tissue samples of PDAC were provided by the Biobank of the Pathology Department after an ethical committee agreement was obtained (P2021/677).

For tumors resected before 2017 (*n =* 18), TNM staging was revised according to the eighth edition of the Union for International Cancer Control (UICC). TRSs were revised by two pathologists for all patients according to the College of American Pathologists (CAP) [34]. Surgical margin assessment was evaluated according to the criteria of the Royal College of Pathologists (RCP) and defined as R1 resection when tumor cells were situated ≤1 mm from surgical or mobilization margins [35]. Resectability status was defined according to the definition of the National Comprehensive Cancer Network (NCCN) [36].

### 2.2. Immunohistochemistry

Standard immunohistochemistry (IHC) was applied to 5-μm-thick sections to display SMAD4 expression (Abcam, Cambridge, UK, Clone EP618Y, dilution 1:150) on FFPE tumor blocks. SMAD4 IHC was performed on DAKO OMNIS using an EnVision/HRP FLEX High pH kit (DAKO Agilent). The sections were counterstained with hematoxylin. For every run, positive and negative controls were performed. Negative controls were carried out by replacing the primary antibody with DAKO Agilent flex antibody diluent, while positive controls consisted of a specimen of normal pancreatic tissue.

### 2.3. Semi-Quantitative Analysis of SMAD4 Expression

SMAD4 nuclear expression was evaluated semi-quantitatively by three independent observers. The slides were revised, and a consensus score was established. SMAD4 loss of expression was defined as <5% of tumor cells with SMAD4 nuclear staining.

### 2.4. Statistical Analysis

Statistical analyses were performed using the STATISTICA software (StatSoft, Tulsa, OK, USA), and graphs were designed using GraphPad Prism version 9.5.1 (GraphPad Software, San Diego, CA, USA). Independent groups of numerical data were compared using the Mann-Whitney U test, while Fisher’s exact test (two groups) or Chi2 test (>2 groups), after verifying application conditions, were employed for categorical data.

Our primary endpoints were OS, progression-free survival (PFS), and metastasis-free survival (MFS). These survival parameters were calculated as the time between the first treatment and the date of last contact or death for OS, the date of recurrence or death for PFS, and metastasis detection or death for MFS. The date of the first treatment was defined as the date of the first cycle of neoadjuvant treatment. The cutoff date for analysis was 17 June 2022. In order to evaluate the difference in survival between groups of patients, a univariate Cox’s model was adopted, including a log-rank test and Kaplan-Meier curves. The Reverse Kaplan-Meier method was used for median follow-up.

Multivariate Cox regression analyses were conducted to identify independent prognostic factors. For multivariate analysis, we selected clinicopathological variables with a *p*-value ≤ 0.1 in univariate analysis in order to build the model according to the rule for at least ten events per variable. We then added SMAD4 status to the most significant clinical model.

We also studied the ability of SMAD4 status to be a predictive biomarker for NAT. Interaction tests between SMAD4 status and the administration of neoadjuvant FOLFIRINOX and Gemcitabine were performed using Kaplan-Meier survival curves with log-rank testing and the multivariate Cox model to calculate the hazard ratios (HRs) with 95% confidence intervals (CIs). Patients receiving other types of NAT than FOLFIRINOX- or Gemcitabine-based were excluded (*n =* 2).

For all tests, a *p*-value < 0.05 was considered statistically significant.

## 3. Results

### 3.1. Study Population

**Figure 1:**
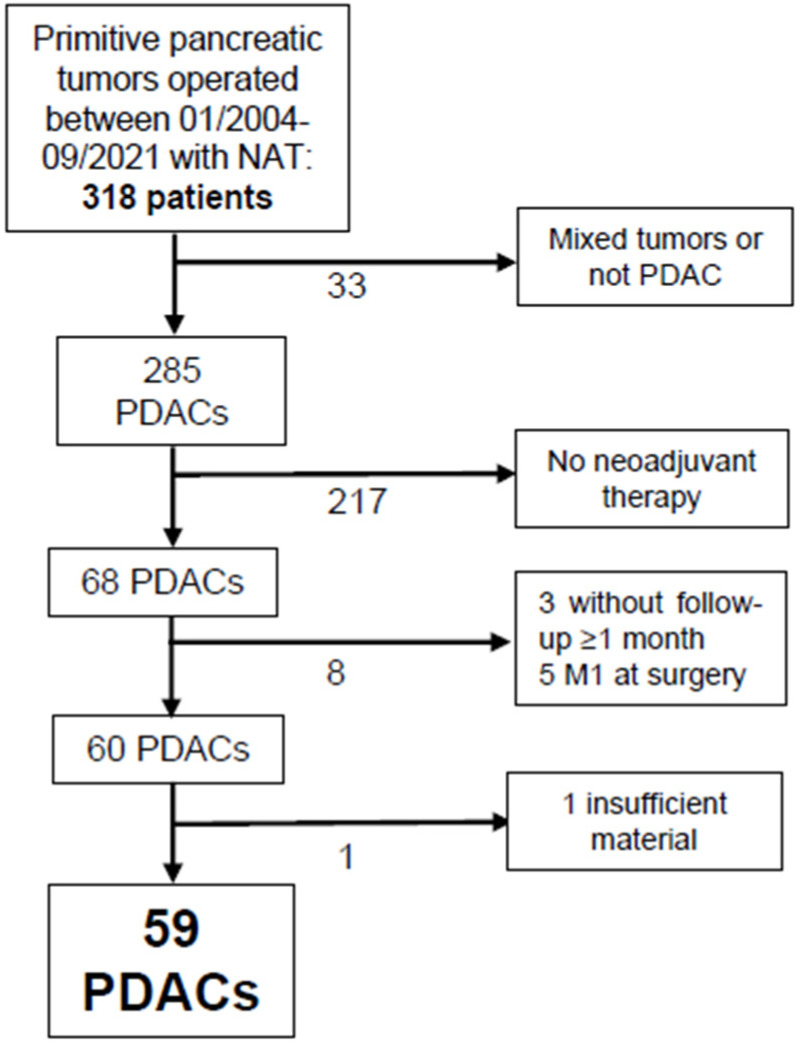
Algorithm of patient selection according to inclusion and exclusion criteria. Abbreviations: PDACs: pancreatic ductal adenocarcinomas; NAT: neoadjuvant therapy.

Between January 2004 and September 2021, 318 patients underwent surgery for a primitive pancreatic tumor in our institution. A total of 33 patients presented a diagnosis other than PDAC, and 217 did not receive NAT prior to surgery. Five patients were metastatic (M1) at the moment of surgery and were therefore excluded. Three other patients were ruled out due to follow-up being shorter than one month, and one patient did not have sufficient residual tumor material to undergo further analysis (Figure 1). We have thus included 59 patients in our study.

The clinical and histopathological characteristics of these patients are summarized in Table 1. Median OS, PFS, and MFS were 24.1, 14.3, and 14.9 months, respectively. The median follow-up was 40.74 months.

The majority of the patients were male, and the mean age of diagnosis was 62 years. Most tumors were borderline resectable at diagnosis (42.37%), and almost a quarter of patients presented resectable tumors.

Half of the patients were operated after 2018, and about 70% received FOLFIRINOX-based NAT. A majority of patients were able to sustain adjuvant therapy after surgery (79.66%), and this therapy was mostly Gemcitabine-based (45.76%).

Regarding histopathological characteristics, the majority of patients presented moderately differentiated tumors (38.98%). The mean tumor size was 3 cm, with almost 50% of patients being staged as pT2. Most patients presented positive LN (59.32%) with a mean positive LN ratio of 10%. Lymphatic, vascular, and perineural invasion was observed in 54.24%, 81.36%, and 79.66% of patients, respectively. Resection margins were more often involved (R1, 74.58%). No cases presented complete tumor regression, and only three patients presented an almost complete response (CAP1 score). The majority of patients presented a partial response (CAP2 score, 64.41%).

### 3.2. SMAD4 Expression Analysis and Association between SMAD4 Status and Clinicopathological Characteristics

**Figure 2:**
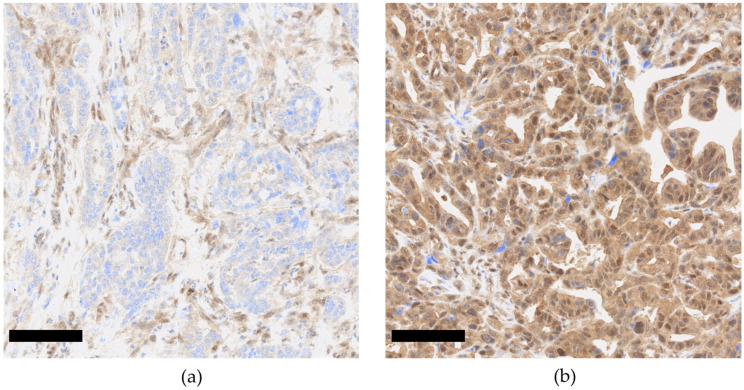
SMAD4 nuclear expression in PDAC (20x, scale bar 100 µm). Loss of SMAD4 nuclear expression in cancer cells (**a**). Maintained SMAD4 expression in cancer cells (**b**).

As many as 49.15% of PDACs presented SMAD4 nuclear loss of expression, defined as the nuclear expression of SMAD4 in <5% of tumor cells (see Section 2) (Figure 2).

The relationship between SMAD4 status and the clinicopathological variables can be found in Table 2.

SMAD4 loss was associated with a higher positive LN ratio (*p* = 0.03). When patients with pN1 and pN2 stage (pN+) were pooled, there was a tendency for patients with SMAD4 negative tumors to present a pN+ stage (*p* = 0.06).

We could not evaluate the association between SMAD4 status and tumor regression scores as the statistical conditions to conduct the statistical test were not met. However, it is interesting to note that the three patients presenting a near-complete response (CAP1 score) all had SMAD4-positive tumors.

### 3.3. The Impact of the Clinicopathological Variables on Outcome

The impact of the different clinicopathological variables on the survival parameters can be found in Table 1.

When univariate analysis was conducted, only tumor size and pT stage influenced OS.

The year of surgery and the type of NAT both played a major role on progression and metastasis in univariate analysis. Indeed, patients receiving Gemcitabine-based therapy presented shorter PFS (HR 2.74 95%CI 1.45–5.19) and MFS (HR 3.01 95%CI 1.58–5.7) compared to those receiving FOLFIRINOX-based regimens. The administration of adjuvant therapy, however, produced no effect on either survival parameter.

Histological grade, tumor size, pN stage, positive LN ratio, and TRS all showed a statistically significant impact on PFS and MFS. However, the presence of vascular invasion was associated with worse PFS but not MFS.

In multivariate analysis, only size was an independent prognostic variable for OS (*p* = 0.002). The type of NAT and pN stage were independent prognostic variables for PFS in multivariate analysis. Concerning MFS, the type of NAT, pN, and pT stage, as well as the TRS, were independent markers (Table S1).

### 3.4. The Impact of SMAD4 Status on Outcome

**Figure 3:**
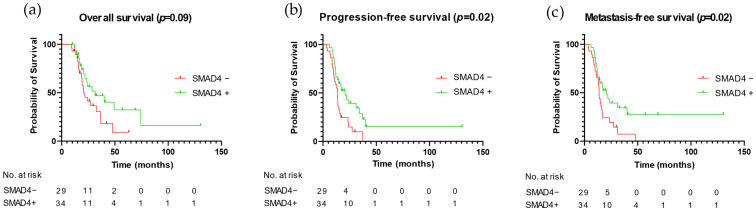
Prognostic impact of SMAD4 on OS (**a**), PFS (**b**), and MFS (**c**). Abbreviations: OS: overall survival; PFS: progression-free survival; MFS: metastasis-free survival.

In univariate analysis, there was a tendency for SMAD4 loss to be associated with worse OS (*p* = 0.09), but SMAD4 negative tumors presented shorter PFS (*p* = 0.02) and MFS (*p* = 0.02) (Figure 3).

However, in multivariate analysis, SMAD4 did not emerge as an independent prognostic variable for either PFS or MFS (Table 3). Indeed, only the type of NAT and the lymph node status were independent prognostic variables for both PFS and MFS.

### 3.5. Interaction Tests between SMAD4 Status and NAT

**Figure 4:**
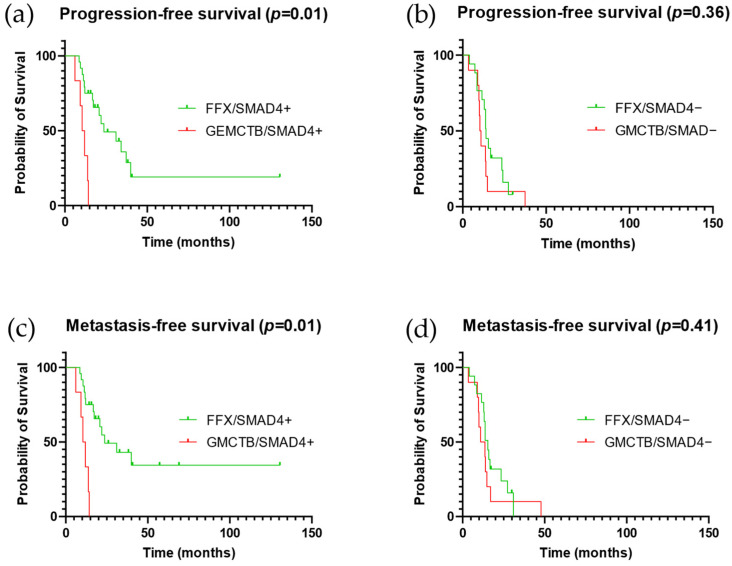
Survival analysis in subgroups of patients determined by the type of administered NAT and SMAD4 status. Comparison among patients with SMAD4 positive tumors (**a**–**c**). Comparison among patients with SMAD4 negative tumors (**b**–**d**). Abbreviations: NAT: neoadjuvant therapy; FFX: FOLFIRINOX; GMCTB: Gemcitabine.

In order to evaluate the predictive effect of SMAD4 status on the type of NAT, we conducted interaction testing (Figure 4 and Table 4).

We observed that patients receiving FOLFIRINOX-based NAT present longer PFS (*p* = 0.01) and MFS (*p* = 0.01) than compared to patients receiving Gemcitabine if the PDAC is SMAD4 positive. In contrast, if the tumor displays SMAD4 loss of expression, we did not observe a statistically significant difference in terms of survival between Gemcitabine- and FOLFIRINOX-based NAT (Figure 4). The best outcome was observed for patients receiving FOLFIRINOX-based NAT with SMAD4-positive tumors (Figure S1).

Based on interaction testing, we suggested that administering FOLFIRINOX to patients with SMAD4-negative tumors was still superior to Gemcitabine treatment but remained non-significant (Table 4). This finding was supported by multivariate analysis. Indeed, FOLFIRINOX-based NAT produced a statistically significant effect on PFS and MFS for patients with SMAD4- positive and negative tumors (Table 5).

## 4. Discussion

NAT is increasingly administered to PDAC patients in hopes of improving resections and increasing survival. However, complete tumor response is rarely achieved. Therefore, reliable biomarkers that can predict responses to NAT in PDAC are needed. In our study, we aimed to analyze the impact of SMAD4 status on the outcomes of patients receiving NAT prior to surgery. In the present study, we observed that SMAD4 loss of expression was associated with worse PFS and MFS. These results corroborate previous results from the few available clinical studies focusing on the prognostic impact of SMAD4 status in a neoadjuvant setting. In the retrospective study of Kadera et al., SMAD4 protein loss was associated with shorter OS and was the only independent prognostic biomarker in patients with LAPC and BRPC who underwent surgery after NAT [32]. In a multi-institutional study including 147 PDAC patients receiving NAT, SMAD4 loss was associated with shorter DFS [37]. Another recent study demonstrated that SMAD4-negative tumors presented worse TRS and treatment response; however, no data on survival was available [30]. In our study, we could not analyze the association between TRS and SMAD4 status due to the small number of patients (*n =* 3) who presented almost complete tumor response (CAP1 score). Nevertheless, it is worth mentioning that all the patients exhibiting major tumor regression displayed SMAD4-positive tumors. However, SMAD4 status did not emerge as an independent prognostic variable in our multivariate model in patients receiving NAT.

SMAD4 loss has been correlated with increased metastatic burden in the clinical setting [9,24,26]. This hypothesis has been supported by in vitro and in vivo studies that have suggested that SMAD4 loss might enhance metastatic potential by inducing specific types of cell migration that facilitate metastasis and neoplastic cell survival [38,39]. We observed that SMAD4 loss was associated with a worse positive LN ratio and shorter MFS in patients receiving NAT, suggesting a higher potential to disseminate. This finding could indicate that SMAD4-negative tumor cells might be more prone to metastasize through hematogenous and lymphatic pathways despite NAT, supporting the idea that patients with SMAD4 loss are more at risk for treatment failure and recurrence. Therefore, patients presenting SMAD4-positive tumors may be more inclined to respond to therapy.

Using interaction testing, we sought the predictive effect of SMAD4 status in patients receiving NAT. Patients receiving FOLFIRINOX-based therapy with SMAD4-positive tumors emerged as having the best outcomes, characterized by longer PFS and MFS. We observed in univariate analysis that SMAD4 status has a predictive effect for FOLFIRINOX-based NAT. Indeed, if patients presented SMAD4-positive PDACs, we observed a significant difference in terms of PFS and MFS between patients receiving FOLFIRINOX- or Gemcitabine-based NAT. In contrast, there was no significant difference between the two groups if the tumor presented a loss of SMAD4 expression. However, the predictive effect is reduced in multivariate analysis. This finding could suggest a potential benefit in SMAD4 status analysis in PDAC patients receiving NAT, as it may allow patient stratification and optimization of treatment. It would, therefore, also be interesting to study SMAD4 expression on biopsy and cytology. Previous studies have demonstrated that SMAD4 may be a potential predictive biomarker in the non-NAT setting and that there is a good concordance between pre-operative and post-operative specimens [40,41,42,43,44]. We had envisioned analyzing SMAD4 expression on biopsy or fine-needle aspiration specimens (FNA). Unfortunately, we were unable to retrieve data for enough patients with biopsy or FNA samples in our cohort to obtain statistically significant results.

Moreover, the group of patients with Gemcitabine-based therapy was relatively small (*n =* 16), and a larger sample size would be needed to confirm this trend. Moreover, Gemcitabine is usually administered to patients with worse performance status as it is associated with fewer secondary effects than FOLFIRINOX.

Studies have suggested that the loss of SMAD4 expression may confer chemoresistance [28]. However, evaluating chemosensitivity is much more complex for combinations of chemotherapy regimens, such as FOLFIRINOX-based therapy, with a severe lack of reliable models [45]. In line with our data, preliminary clinical and molecular data from the Know Your Tumor (KYT) pancreas cancer program, which enables multiomic testing for DAC patients, suggests that low expression of SMAD4 and its target genes may contribute to FOLFIRINOX resistance [46,47]. In a recent study where the impact of driver alterations in the progression and response of PDAC patients under FOLFIRINOX-based treatment was analyzed, SMAD4 alterations were associated with more frequent treatment failure and metastatic progression under NAT [31]. Further translational investigations are needed to improve our understanding of the underlying mechanisms of SMAD4 inactivation-induced chemoresistance.

The weakness of our study is linked to the inherent limitations of retrospective research [33,48]. Our study included a relatively small sample of patients (*n =* 59). Moreover, one limitation is related to the long study period. Indeed, surgical techniques, histological analysis (in particular margin assessment), and therapy have evolved and changed over time, which may bias our results. However, we addressed this issue by including the year of surgery in our univariate and multivariate analyses. We demonstrated that the more recent the surgery, and therefore the more recent the patient care, the longer the PFS and MFS are. Nevertheless, OS remained unaffected. Moreover, the year of surgery did not emerge as an independent prognostic factor in multivariate analysis.

## 5. Conclusions

In conclusion, NAT is increasingly used in the treatment of PDAC patients with the aim of improving resection and increasing survival, but reliable biomarkers are needed to predict response to therapy. Our study showed that patients with SMAD4 loss may be associated with a higher and quicker potential for dissemination and that patients with SMAD4-positive tumors may be better candidates for FOLFIRINOX-based NAT.

Larger prospective studies are needed, and the identification and characterization of SMAD4 molecular alterations might improve our understanding of the underlying mechanisms of SMAD4 inactivation-induced chemoresistance and increased metastatic burden. Research on biopsy and cytology specimens would be of particular interest to study SMAD4’s potential as a predictive biomarker.

## Figures and Tables

**Table 1:** Impact of clinicopathological factors on overall survival (OS), progression-free survival (PFS), and metastasis-free survival (MFS).

		OS		PFS		MFS	
Variables	*n* = 59	HR (95% CI)	*p*-Value	HR (95% CI)	*p*-Value	HR (95% CI)	*p*-Value
**Sex, *n* (%)**							
Male	37 (62.71%)	1		1		1	
Female	22 (37.29%)	0.52 (0.25–1.07)	0.06	0.63(0.34–1.18)	0.14	0.70 (0.37–1.31)	0.25
Age, mean (range), y	62 (41–79)	0.99 (0.96–1.03)	0.87 ^1^	1.02 (0.99–1.05)	0.31 ^1^	1.01 (0.98–1.04)	0.41 ^1^
CA19.9, mean (range), U/mL	2437 (0–40,739)	0.99 (0.99–1)	0.27 ^1^	0.99 (0.99–1.00002)	0.26 ^1^	0.99 (0.99–1)	0.44 ^1^
Year of surgery, median (range)	2018 (2005–2021)	0.99 (0.99–1)	0.35 ^1^	0.99 (0.9993–0.9998)	**0.001 ^1^**	0.99 (0.9994–0.9999)	**0.004 ^1^**
Resectability, *n* (%)							
Resectable	14 (23.73%)	1		1		1	
Borderline	25 (42.37%)	1.19 (0.46–3.05)		0.80 (0.35–1.82)		0.78 (0.34–1.78)	
Locally advanced	17 (28.81%)	0.83 (0.32–2.19)		0.86 (0.37–2.02)		0.87 (0.37–2.04)	
Metastatic	2 (3.39%)	3.52 (0.58–21.40)		1.22 (0.26–5.69)		1.29 (0.27–6.05)	
Unknown	1 (1.69%)		0.68		0.81		0.82
Type of neoadjuvant therapy, *n* (%)							
FOLFIRINOX-based	41 (69.49%)	1		1		1	
Gemcitabine-based	16 (27.12%)	1.6 (0.80–3.20)		3.06 (1.61–5.81)		3.01 (1.58–5.7)	
Other	2 (3.39%)		0.28		**0.007**		**0.008**
Adjuvant therapy, *n* (%)							
No	7 (11.86%)	1		1		1	
Yes	47 (79.66%)	0.67 (0.25–1.76)		1.04 (0.44–2.48)		1.29 (0.27–6.04)	
Unknown	5 (8.47%)		0.45		0.92		0.57
Type of adjuvant therapy, *n* (%)							
Gemcitabine	27 (45.76%)	1		1		1	
FOLFIRINOX	18 (30.51%)	0.72 (0.31–1.63)		0.71 (0.37–1.45)		0.75 (0.37–1.51)	
Other	1 (1.69%)						
Unknown	1 (1.69%)		0.22		0.28		0.36
Histologic grade, *n* (%)							
Well-differentiated (G1)	12 (20.34%)	1		1		1	
Moderately differentiated (G2)	23 (38.98%)	0.45 (0.50–1.47)		1.99 (0.93–4.77)		2.88 (1.07–7.78)	
Poorly differentiated (G3)	17 (28.81%)	2.88 (0.91–9.08)		2.07 (0.82–5.24)		2.93 (1.05–8.21)	
Unknown	7 (11.86%)		0.15		**0.03**		**0.04**
Size, mean (range), mm	33 (6–80)	1.03 (1.01–1.05)	**0.002 ^1^**	1.02 (1.005–1.04)	**0.01 ^1^**	1.02 (1.006–1.04)	**0.007 ^1^**
pT, n (%)							
pT1a	0 (0%)						
pT1b	2 (3.39%)						
pT1c	10 (16.95%)	1		1		1	
pT2	29(49.15%)						
pT3	17 (28.81%)	2.49 (0.89–6.94)		2.11 (0.89–4.96)		2.39 (0.94–6.02)	
pT4	1 (1.69%)	6.23 (1.74–22.20)	**0.04 ^2^**	2.79 (1.13–6.90)	0.06 ^2^	3.78 (1.36–10.50)	0.06 ^2^
pN, *n* (%)							
pN0	24 (40.68%)	1		1		1	
pN+	35 (59.32%)						
pN1	20 (33.90%)	1.68 (0.78–3.59)		1.50 (0.74–3.06)		1.44 (0.70–3.002)	
pN2	15 (25.42%)	1.64 (0.67–3.98)	0.28	3.16 (1.49–6.70)	**0.02**	2.96 (1.40–6.28)	**0.04**
Positive LN ratio, mean (range)	0.10 (0–0.63)	10.08 (0.86–118.04)	0.09 ^1^	252.42 (12.78–4986.16)	**0.003 ^1^**	275.36 (13.42–5649.54)	**0.0003 ^1^**
Lymphatic invasion, *n* (%)							
Negative	27 (45.76%)	1		1		1	
Positive	32 (54.24%)	1.39 (0.71–2.71)	0.33	1.68 (0.94–3.02)	0.08	1.61 (0.89–2.92)	0.12
Vascular invasion, *n* (%)							
Negative	11 (18.64%)	1		1		1	
Positive	48 (81.36%)	1.47 (0.66–3.26)	0.36	2.75 (1.37–5.50)	**0.02**	1.99 (0.97–4.06)	0.09
Perineural invasion, *n* (%)							
Negative	12 (20.34%)	1		1		1	
Positive	47 (79.66%)	1.004 (0.45–2.22)	0.99	1.24 (0.59–2.60)	0.54	1.46 (0.68–3.15)	0.29
Resection margin (RCP), *n* (%)							
Negative	15 (25.42%)	1		1		1	
Positive	44 (74.58%)	1.16 (0.52–2.58)	0.71	1.56 (0.75–3.24)	0.22	1.84 (0.85–3.97)	0.1
Histologic tumor regression score (CAP), *n* (%)							
Complete response (score 0)	0 (0%)	1		1		1	
Near complete response (score 1)	3 (5.08%)	1.001 (0.23–4.30)		1.02 (0.24–4.39)		1.01 (0.24–4.33)	
Partial response (score 2)	38 (64.41%)	1.32 (0.28–6.28)		2.30 (0.52–10.19)		2.23 (0.50–9.82)	
Poor or no response (score 3)	18 (30.51%)		0.43		**0.02**		**0.03**

^1^ Survival data were analyzed using a univariate COX regression model, and *p*-values were obtained using the Kaplan-Meier method with log-rank testing except for continuous variables where univariate Cox regression model was used; ^2^ pT1b and pT1c tumors and pT3 and pT4 tumors were pooled for the analysis. All “unknown data” were excluded from the analysis. Abbreviations: NAT: neoadjuvant therapy; OS: overall survival; PFS: progression-free survival; MFS: metastasis-free survival; HR: hazard ratio; CI: confidence interval; LN: lymph node, RCP: Royal College of Pathologists; CAP: College of American pathologists.

**Table 2:** Relationship between SMAD4 status and clinicopathological variables.

Variables	SMAD4 −	SMAD4 +	Fisher’s Exact Test or Chi^2^ Test (except ^n^)
	(*n* = 29)	(*n* = 30)	
**Sex, *n* (%)**			
Female	10 (34.48%)	12 (40%)	
Male	19 (65.52%)	18 (60%)	0.79
Age, mean (range), y			
	64 (41–79)	60 (42–75)	0.73 ^1^
Size, mean (range), mm			
	36 (12–80)	31 (6–64)	0.50 ^1^
CA19.9, mean (range), U/mL			
	1243.39 (1.91–7977)	3488.36 (0–40,738.80)	0.17 ^1^
Histologic grade, *n* (%)			
Well-differentiated (G1)	5 (17.24%)	7 (23.33%)	
Moderately differentiated (G2)	13 (44.83%)	10 (33.33%)	
Poorly differentiated (G3)	8 (27.59%)	9 (30%)	
Unknown	3 (10.34%)	4 (13.33%)	0.68
pT, *n* (%)			
pT1b	0 (0%)	2 (6.67%)	
pT1c	5 (17.24%)	5 (16.67%)	
pT2	15 (51.72%)	14 (46.67%)	
pT3	8 (27.59%)	9 (30%)	
pT4	1 (3.45%)	0 (0%)	
Unknown	0 (0%)	0 (0%)	0.75 ^2^
pN, *n* (%)			
pN0	8 (27.59%)	16 (53.33%)	
pN+	21 (72.41%)	14 (46.67%)	
pN1	11(37.93%)	9 (30%)	
pN2	10 (34.48%)	5 (16.67%)	
Unknown	0 (0%)	0 (0%)	0.10
Positive LN ratio, mean (range)	0.13 (0–0.65)	0.07 (0–0.35)	**0.03 ^1^**
Lymphatic invasion, *n* (%)			
Positive	16 (55.17%)	11 (36.67%)	
Negative	13 (44.83%)	19 (63.33%)	0.19
Vascular invasion, *n* (%)			
Positive	7 (24.14%)	4 (13.33%)	
Negative	22 (75.86%)	26 (86.67%)	0.33
Perineural invasion, *n* (%)			
Positive	24 (82.76%)	23 (76.67%)	
Negative	5 (17.24%)	7 (23.33%)	0.75
Resection margin (RCP), *n* (%)			
Positive	22 (75.86%)	22 (73.33%)	
Negative	7 (24.14%)	8 (26.67%)	>0.99
Histologic tumor regression score (CAP), *n* (%)			
Complete response (score 0)	0 (0%)	0 (0%)	
Near complete response (score 1)	0 (0%)	3 (10.00%)	
Partial response (score 2)	18 (62.07%)	20 (66.67%)	
Poor or no response (score 3)	11 (37.93%)	7 (23.33%)	NA ^3^

^1^ Mann-Whitney test for independent data groups; ^2^ pT1b and pT1c tumors and pT2-pT4 tumors were pooled for the analysis ^3^ Chi^2^ calculations are not valid because the application conditions are not met (all expected values must be greater than 1.0, and at least 20% of the expected values must be greater than 5). All “unknown data” was excluded from the analysis. Abbreviations: LN: lymph node; RCP: Royal College of Pathologists; CAP: College of American Pathologists; NA: not applicable.

**Table 3:** Multivariate analysis for OS, PFS, and MFS.

Model *p*-Value	Variables	HR	95% CI	*p*-Value
Overall survival *p* = 0.003	Sex (male vs. female) ^1^	0.50	[0.24–1.05]	0.07
	Tumor size (in mm)	1.03	[1.009–1.05]	**0.004**
	SMAD4 status ^2^	0.62	[0.30–1.26]	0.19
Progression-free survival *p* < 0.00001	Type of NAT (FOLFIRINOX-based vs. Gemcitabine based) ^3^	7.70	[3.16–18.77]	**0.000007**
	pN (pN0–1 vs. pN2) ^4^	2.90	[1.35–6.20]	**0.006**
	Histologic grade (G1–2 vs. G3) ^5^	1.55	[0.78–3.09]	0.21
	Tumor regression score (CAP score 1–2 vs. 3) ^6^	1.80	[0.91–3.56]	0.09
	SMAD4 status ^2^	0.72	[0.35–1.50]	0.37
Metastasis-free survival *p* = 0.00001	Type of NAT (FOLFIRINOX-based vs. Gemcitabine based) ^3^	5.49	[2.44–12.31]	**0.00004**
	pN (pN0–1 vs. pN2) ^4^	3.43	[1.59–7.41]	**0.002**
	pT (pT1 vs. pT2-3-4) ^7^	4.67	[1.45–15.05]	**0.01**
	Tumor regression score (CAP score 1 vs. 2–3) ^8^	0.13	[0.02–0.95]	**0.04**
	SMAD4 status ^2^	0.87	[0.44–1.74]	0.71

^1^ Male patients were used as reference; ^2^ patients with SMAD4 loss of expression were used as reference; ^3^ patients who received FOLFIRINOX-based NAT were used as reference; ^4^ patients with pN0–1 stage were used as reference; ^5^ patients with G1–2 histologic grade were used as reference; ^6^ patients with a tumor regression score of CAP1–2 were used as reference; ^7^ patients with pT1 stage were used as reference; ^8^ patients with a tumor regression score of CAP1–2 were used as reference. Abbreviations: OS: overall survival, FS: disease-free survival; MFS: metastasis-free survival; NAT: neoadjuvant therapy; HR: hazard ratio; CI: confidence interval; CAP: College of American Pathologists.

**Table 4:** Interaction test between SMAD4 status and the type of NAT.

Model *p*-Value	Variables	HR	95% CI
Progression-free survival *p* = 0.001	Gemcitabine-based NAT/SMAD4−	1	
	Gemcitabine-based NAT/SMAD4+	1.60	[0.57–4.43]
	FOLFIRINOX-based NAT/SMAD4+	**0.27**	[0.12–0.60]
	FOLFIRINOX-based NAT/SMAD4−	0.68	[0.30–1.53]
Metastasis-free survival *p* = 0.0008	Gemcitabine-based NAT/SMAD4−	1	
	Gemcitabine-based NAT/SMAD4+	1.86	[0.67–5.20]
	FOLFIRINOX-based NAT/SMAD4+	**0.27**	[0.12–0.61]
	FOLFIRINOX-based NAT/SMAD4−	0.71	[0.31–1.58]

Abbreviations: NAT: neoadjuvant therapy; HR: hazard ratio; CI: confidence interval.

**Table 5:** Interaction test between SMAD4 status and type of NAT in the most significative clinical model.

Model *p*-Value	Variables	HR	95% CI	*p*-Value
Disease-free survival *p* < 0.00001	Gemcitabine-based NAT/SMAD4−	1		
	Gemcitabine-based NAT/SMAD4+	1.31	[0.46–3.71]	0.61
	FOLFIRINOX-based NAT/SMAD4+	0.10	[0.03–0.29]	**0.00002**
	FOLFIRINOX-based NAT/SMAD4−	0.21	[0.07–0.60]	**0.004**
	pN (pN0–1 vs. pN2) ^1^	2.65	[1.23–5.72]	**0.01**
	Histologic grade (G1–2 vs. G2) ^2^	1.43	[0.71–2.86]	0.31
	Tumor regression score (CAP score 1–2 vs. 3) ^3^	2.21	[1.11–4.38]	**0.02**
Metastasis-free survival *p* = 0.00001	Gemcitabine-based NAT/SMAD4−	1		
	Gemcitabine-based NAT/SMAD4+	1.02	[0.35–2.95]	0.97
	FOLFIRINOX-based NAT/SMAD4+	0.15	[0.06–0.39]	**0.00009**
	FOLFIRINOX-based NAT/SMAD4−	0.21	[0.07–0.61]	**0.004**
	pN (pN0–1 vs. pN2) ^1^	3.11	[1.36–7.11]	**0.007**
	pT (pT1 vs. pT2-3-4) ^4^	5.07	[1.52–16.92]	**0.008**
	Tumor regression score (CAP score 1 vs. 2–3) ^5^	0.11	[0.02–0.83]	**0.03**

^1^ Patients with pN0-1 stage were used as reference; ^2^ patients with G1–2 histologic grade were used as reference; ^3^ patients with a tumor regression score of CAP1–2 were used as reference; ^4^ patients with pT1 stage were used as reference; ^5^ patients with a tumor regression score of CAP1–2 were used as reference. Abbreviations: NAT: neoadjuvant therapy; HR: hazard ratio; CI: confidence interval; LN: lymph node, CAP: College of American Pathologists.

## Data Availability

The data presented in this study are available on request from the corresponding author.

## Supplementary Materials

The following supporting information can be downloaded at: https://www.mdpi.com/article/10.3390/cancers15153765/s1, Figure S1: Survival analysis in subgroups of patients determined by the type of administered NAT and SMAD4 status; Table S1: Multivariate analysis for PFS and MFS.
