# Supplementary material for: SMAD4 Positive Pancreatic Ductal Adenocarcinomas Are Associated with Better Outcomes in Patients Receiving FOLFIRINOX-Based Neoadjuvant Therapy
Source: Cancers (Basel). 2023 Jul 25;15(15):3765. doi: 10.3390/cancers15153765 (PMC10417261; doi:10.3390/cancers15153765)
Supplement: Supplementary file 1 [file cancers-15-03765-s001.zip › cancers-2503417-supplementary.pdf]

Table S1. Multivariate analysis for PFS and MFS

Model p-value	Variables	HR	95% CI	P-value
**Overall survival p=0.002**	Sex (male vs female)[1]	0.49	[0.23-1.02]	0.06
	Tumor size (in mm)	1.03	[1.01-1.05]	**0.002**
**Progression-free survival p<0.00001**	Type of NAT (FOLFIRINOX-based vs Gemcitabine based)[2]	8.43	[3.53-20.15]	**0.00002**
	pN (pN0-1 vs pN2)[3]	3.22	[1.56-6.62]	**0.0014**
	Histologic grade (G1-2 vs G3)[4]	1.61	[0.81-3.19]	0.17
	Tumor regression score (CAP score 1-2 vs 3)[5]	1.82	[0.92-3.57]	0.083
**Metastasis-free survival p<0.00001**	Type of NAT (FOLFIRINOX-based vs Gemcitabine based)[2]	5.69	[2.61-12.44]	**0.000013**
	pN (pN0-1 vs pN2)[3]	3.61	[1.76-7.42]	**0.00047**
	pT (pT1 vs pT2-3-4)[6]	4.56	[1.43-14.52]	**0.01**
	Tumor regression score (CAP score 1 vs 2-3)[7]	0.13	[0.02-0.95]	**0.047**

[1]Male patients were used as reference; [2]Patients who received FOLFIRINOX-based NAT are used as reference; [3]Patients with pN0-1 stage were used as reference; [4]Patients with G1-2 histologic grade were used as reference; [5]Patients with a tumor regression score of CAP1-2 were used as reference; [6]Patients with pT1 stage were used as reference; [7]Patients with a tumor regression score of CAP1 were used as reference

Abbreviations: OS: Overall survival, PFS: Disease-free survival; MFS: Metastasis-free survival; NAT: Neoadjuvant therapy; HR : Hazard Ratio; CI: Confidence interval; CAP : College of American Pathologists.

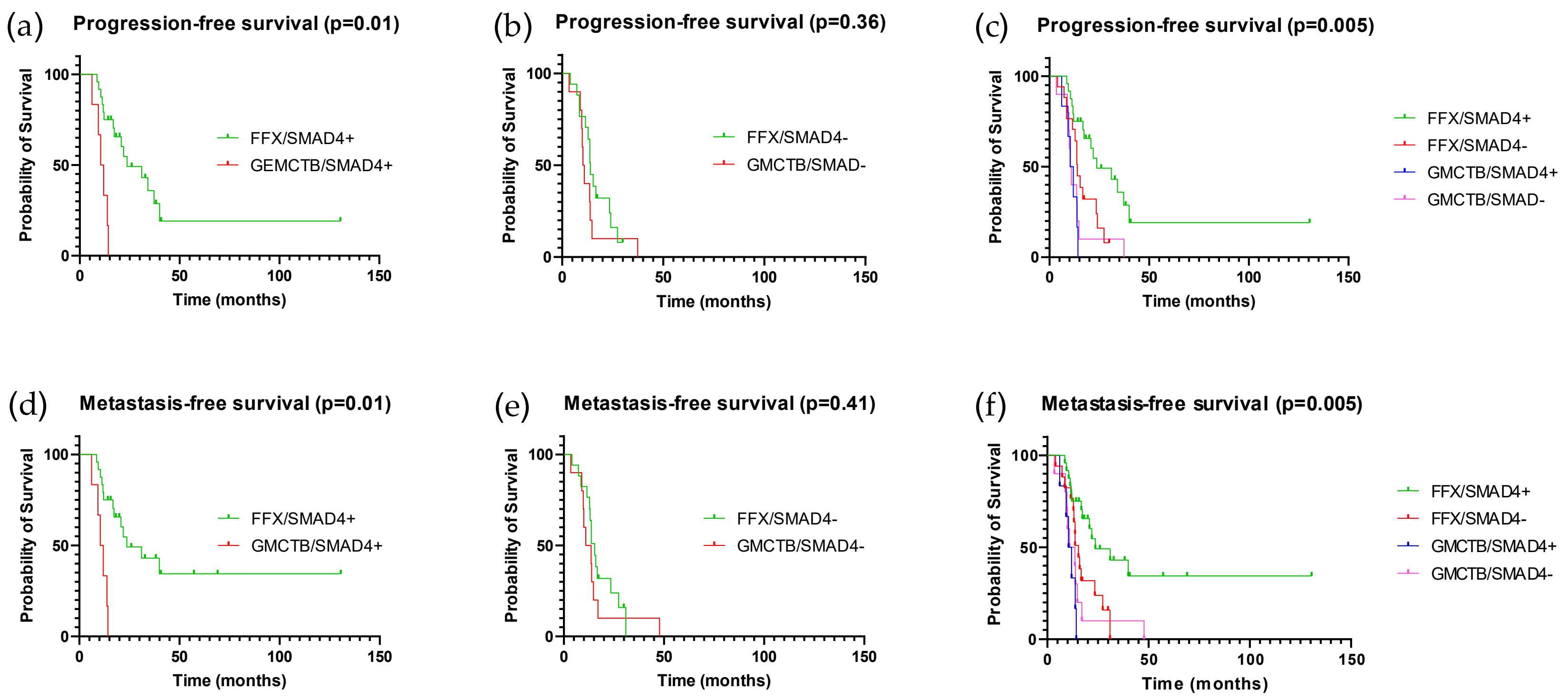

Figure S1. Survival analysis in subgroups of patients determined by the type of administered NAT and SMAD4 status. Abbreviations : NAT: Neoadjuvant therapy; FFX: FOLFIRINOX; GMCTB: Gemcitabine
